# Morphometric Characteristics and Genetic Issr Marker Variability in *Rhodiola rosea* L. (Crassulaceae) in Different Ecological and Geographic Conditions in the Altai Republic

**DOI:** 10.3390/ijms242015224

**Published:** 2023-10-16

**Authors:** Olga V. Dorogina, Irina N. Kuban, Altynai A. Achimova, Natasha Williams, Nicolay N. Lashchinskiy, Elena V. Zhmud

**Affiliations:** 1 Central Siberian Botanical Garden SB RAS, Zolotodolinskaya Str. 101, Novosibirsk 630090, Russia; 2 Gorno-Altai Botanical Garden, Chisty Lug, Shebalinsky District, Kamlak 649218, Russia; 3 Department of Food Science and Human Nutrition, Colorado State University, 502 W Lake St., Fort Collins, CO 80523, USA; 4 NSU Climate Center, Novosibirsk State University, Pirogova Str. 1, Novosibirsk 630090, Russia

**Keywords:** protected areas, cenopopulations, rare (“Red Book”) species, comparative analysis of morphometric parameters, intraspecific genetic polymorphism, parameters of genetic diversity, anthropogenic stress

## Abstract

*Rhodiola rosea* L. is a vulnerable species in the Altai Republic (AR) and Russia in general. For the first time on the territory of AR, studies of the adaptive capabilities of the species and genetic differentiation using ISSR markers were carried out in seven cenopopulations (CP) of *R. rosea* in 2018 and 2020. The research was founded on the notion of conducting a comparative analysis of the morphogenetic structure of *Rhodiola rosea* populations in various ecological and geographical conditions of AR. The aim of this work is to evaluate the variability of morphometric traits of sexually mature living female *R. rosea* plants and to conduct a comparative analysis of genetic variability in cenopopulations (CP) both under undisturbed conditions and under stressful conditions of anthropogenic impact (grazing). Of the 8 primers used, HB12 turned out to be the most informative. The percentage of polymorphic loci in the populations between 0 and 88%. Two populations, located in favorable conditions at relatively low absolute altitudes (2000 m above sea level) (masl) in the undisturbed habitats of the Katun and Altai reserves of AR, were characterized by higher polymorphism. The share of polymorphic loci reached 80%. According to the analysis of statistical data, the highest values of morphometric parameters of the aerial parts of *R. rosea* plants and the highest potential seed productivity were also recorded in these habitats. Representatives of two high-mountain CPs (2400–2500 masl) in the Sailyugemsky National Park (SNP) were characterized by the lowest genetic polymorphism. Their genetic structure is the most homogeneous, since we have not found polymorphic loci. Due to spatial isolation, these individuals are reliably genetically differentiated. In addition, individuals of one type were subjected to stressful anthropogenic impact (grazing). Therefore, the smallest sizes and lowest potential seed productivity were recorded. Our research shows that alpine populations of *R. rosea* in AR, under conditions of anthropogenic stress, need protection for their gene pool.

## 1. Introduction

*Rhodiola rosea* L. (Crassulaceae) is an herbaceous polycarpic plant belonging to long-shoot hemicryptophytes. It is a dioecious species with separate female and male plants. The flowers are collected in a cymose inflorescence; the carpels of the ovary are free and isomeric [1,2]. In the “Red Books” of the Russian Federation [3] and of the Altai Republic (AR) [4], the species is listed as vulnerable and has a resource value [3]. The species occupies a wide disparate hypoarctic Eurasian–American circumboreal range in the northern hemisphere. This species originated in the Qinghai–Tibet Plateau, from which it migrated west along the Ural Mountains, east along the mountain ranges of eastern Siberia, and northwest into the Taimyr–Siberian region. In Europe, *R. rosea* is common in Iceland, the British Isles, the European Alpine system, and Scandinavia. Also, the species is common in western and eastern North America, up to the eastern coastal regions. It has a narrow area of arcto-alpine habitats [5].

*R. rosea* in southern Siberia (Russia) is distributed in mountainous regions: Tuva, eastern and western Sayans, Kuznetsk Alatau, and Mountain Altai. According to the published data, the main features of the morphostructure of the aboveground part of the shoots of *R. rosea* have a wide variability that depends on environmental conditions, including the length of the shoots, the size of the leaves, and the number of flowers. This species is a valuable medicinal plant since the rhizomes of *R. rosea* are traditionally used for medicinal purposes [1,6]. The beneficial properties of this species are described in a large number of literary sources, accounting for more than 500 publications in the last 5 years (2018–2023) [7,8].

A sharp decline in the abundance of *R. rosea* in its natural habitat has occurred over the past few years due to excessive harvesting of rhizomes. This is one of the evident examples of the negative impact of the anthropogenic factor on the environment and the overall state of a particular species, which is rare at present due to excessive exploitation of natural resources [9].

In relation to the conservation issue of *R. rosea*, it is necessary to study the state of the species’ coenopopulations within its natural range. Assessment of the biological features, ontogenetic, ecological, and phytocenotic structure of *R. rosea* populations has previously been conducted in the southern Urals and in the highlands of eastern Kazakhstan (EK) [10,11]. However, we are presenting the first study of the variability of the morphostructure traits of the aboveground part of the mature plants of this species in natural cenopopulations (CP) in the Altai Republic.

Molecular markers have a wide potential for the detection of intra- and interpopulation genetic variability in different species (plants, fungi, etc.) [12,13,14]. The genetic structure of *R. rosea* has been studied in the populations of this species in northern Europe [15]. The issues of intrageneric and interspecific phylogenetic relationships of the genus *Rhodiola* have been discussed for the territories of western Europe and eastern Asia [16,17,18,19]. Interspecific variability of the genetic structure was studied for three species of *Rhodiola* in eastern Kazakhstan [20]. Up to date, there have been no studies of inter- and intrapopulation genetic diversity in the populations of this rare species in the Altai Republic. The basis for the research was the task of conducting a comparative analysis of the morphogenetic structure of *R. rosea* populations in undisturbed habitats and under stressful conditions of anthropogenic impact (grazing).

The purpose of the study is to characterize the variability of morphometric traits and to perform a comparative analysis of the genetic variability of *Rhodiola rosea* in natural cenopopulations in various ecological and geographical conditions of the Altai Republic.

## 2. Results and Discussion

### 2.1. Results of Morphometric Studies

Some biometric parameters in *R. rosea* did not differ in different growing conditions, maintaining stable values in the whole sample. We attributed the leaf width and the ratio of the number of generative to vegetative shoots to such features. The most robust individuals of this species developed under natural conditions in the KNR (in CP 1 and CP 2). The indicators of shoot length, diameter of the aerial part, and number of shoots in an individual were characterized by the highest values in the representatives of these CPs that grew under natural undisturbed conditions (Table 1). Representatives of *R. rosea* in CP 1 had the largest diameter of inflorescence with fruits compared to individuals from all other CPs (Table 2) (*p* = 0.01).

Representatives from CP 7 also grew under natural conditions in a protected area and were characterized by the highest number of shoots per individual, including generative ones, and a smaller length of shoot in comparison to the values of the representatives from the two high-mountain samples CP 3 and CP 6. Representatives from CP 3 at the highest altitude were distinguished by the minimum foliage. Individuals of *R. rosea* from CP 6, which grew in high-mountain location and under conditions of anthropogenic impact (grazing), were characterized by the smallest values of the diameter of the aerial part, leaf length, number of shoots (including vegetative ones), and low leaf area index compared with the plants from other CPs (Table 1).

Intermediate values of most of the studied morphometric traits were found in CP 4 and CP 5, which grew in unprotected areas. The lowest number of shoots in individuals (generative and vegetative) was found in individuals from CP 5 (found in Seminsky Pass). In general, climatic conditions in the habitat of CP 5 are favorable. The relatively low altitude of AR is combined here with the growth of individuals of this species on the banks of streams formed by melting snowfields, that is, in close proximity to running water during the entire growing season. However, growth near popular hiking trails makes unregulated harvesting of rhizomes possible. Also, farm animals occasionally graze in this habitat in the summer. Such an environment could have contributed to the removal of the most productive individuals of *R. rosea* from the population.

Some parameters of potential seed productivity did not differ in representatives of *R. rosea* in different CPs (Table 2). The diameter of the inflorescence with fruits and the number of carpels in the ovary had stable values. Under natural conditions, plants in the KNR (CP 2) and the ANR (CP 7) had the highest number of flowers in inflorescences, in addition to having a significantly higher PSP than the representatives from all other ecological and geographical conditions. This may be due to a significantly greater capacity of inflorescences with fruits (Table 2).

On high mountains and in the presence of grazing, *R. rosea* individuals had the lowest values of PSP (SNP, CP 6). Representatives from this CP are also characterized by the smallest size of several other morphometric characteristics, as mentioned above. The same low PSP was observed in the specimens of this species from Seminsky Pass (CP 5), which also grew under conditions of certain anthropogenic impact (Table 2).

The variability of the studied indicators was categorized as high and very high. The parameters of shoot length and the number of carpels in the ovary showed low variability (Table 1 and Table 2).

The study of correlations between the traits of *R. rosea* revealed 10 pairs of traits that were positively correlated to a significant and strong degree. The diameter of the aerial part determines the length of the shoots (r = 0.7), the size of the inflorescence (r = 0.5), the number of leaves (r = 0.7), and the total number of shoots per individual (r = 0.5), including vegetative ones (r = 0.5). The number of shoots in an individual is mainly determined by the number of vegetative shoots (r = 0.9). The number of generative shoots affects the total number of shoots (r = 0.6) and the ratio of generative to vegetative shoots in individuals (r = 0.6). Wider leaf blades are negatively associated with the leaf index (r = −0.5). PSP is determined by the diameter and number of inflorescences with fruits (r = 0.62 and r = 0.98, respectively).

**Figure 1:**
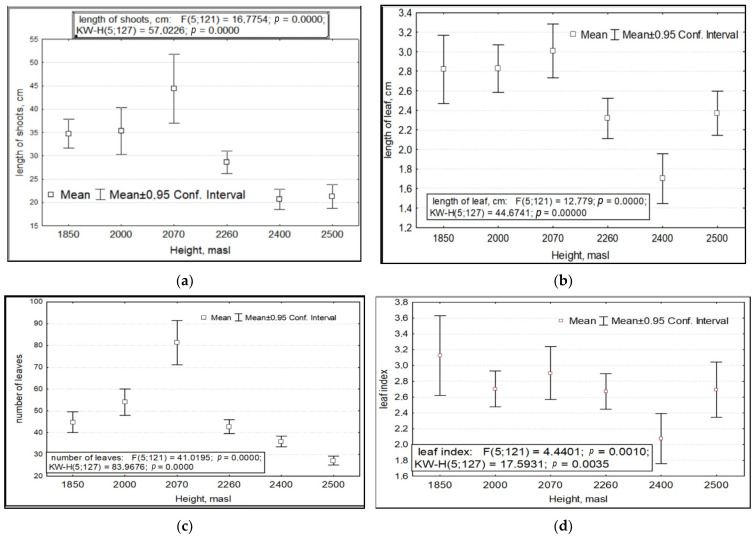

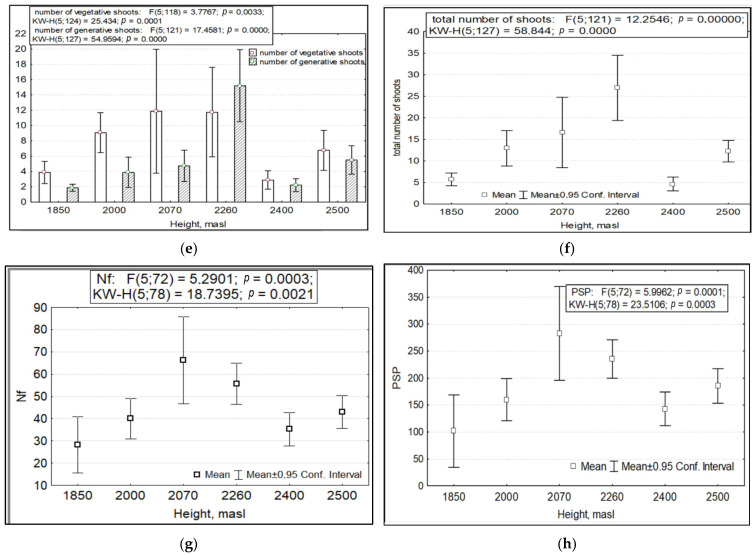
Variability of morphometric features of *Rhodiola rosea* at different absolute altitudes. Designations: (**a**) length of shoots; (**b**) length of leaves; (**c**) number of leaves; (**d**) leaf index: ratio of max to min size; (**e**) number of vegetative and generative shoots; (**f**) total number of shoots; (**g**) number of inflorescences with fruits; (**h**) potential seed productivity. On the *X*-axis—the values of features with a 95% confidence interval; along the Y axis—gradations of the factor.

When considering the variance of the total pooled sample of all *R. rosea* CPs studied, we found that the variability of a number of morphometric characteristics depends on three factors. These are abiotic conditions, which are (1) the absolute altitude of growth and (2) the year of research with its specific growing conditions, and the biotic factor, (3) anthropogenic impact. The parameters of variability of shoot and leaf length, foliage, leaf index, number of generative and vegetative shoots and their sum, number of flowers in inflorescence with fruits, and PSP depended significantly on the absolute altitude of growth (Figure 1).

**Figure 2:**
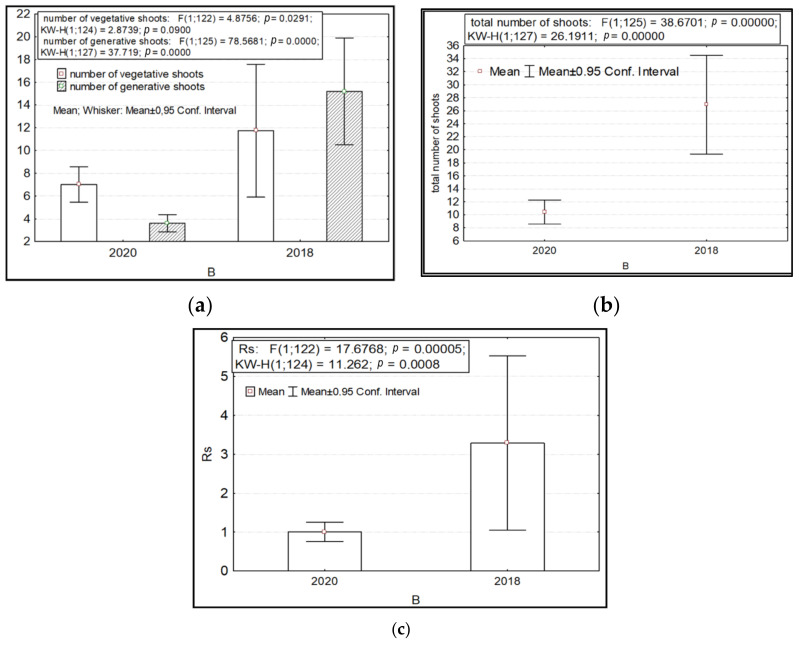
Variability of some morphometric features of *Rhodiola rosea* in different years of research (factor B). Designations: (**a**) number of vegetative and generative shoots; (**b**) total number of shoots; (**c**) the ratio of the number of generative to vegetative shoots. On the *X*-axis—the values of features with a 95% confidence interval; along the Y axis—years of research.

The variability in the number of generative and vegetative shoots and their total number and ratio depended significantly on the year of the study (factor B). Higher values of these parameters were recorded for *R. rosea* in 2018 (Figure 2).

**Figure 3:**
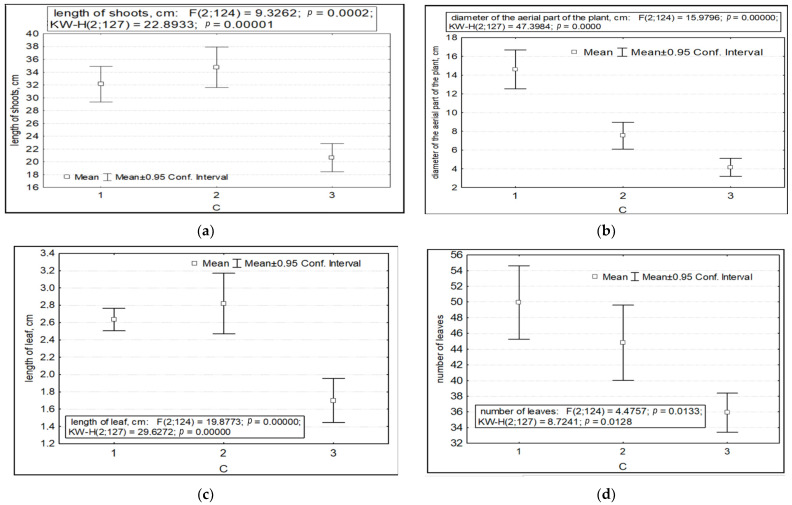

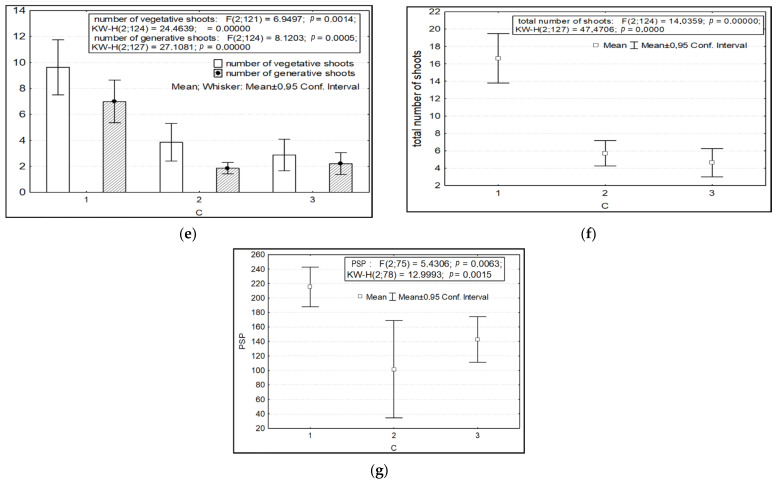
Variability of morphometric characteristics of *Rhodiola rosea* under different anthropogenic impacts (factor C). Designations: (**a**) length of shoots; (**b**) diameter of the aerial part of the plant; (**c**) length of leaves; (**d**) number of leaves; (**e**) number of vegetative and generative shoots; (**f**) total number of shoots; (**g**) potential seed productivity. On the *X*-axis—the values of features with a 95% confidence interval; along the Y axis—gradations of the factor.

The parameters of variability in the length of shoots, the diameter of the aerial parts of plants, the length and number of leaves, the number of shoots (both vegetative and generative), and potential seed productivity depended on the factor of anthropogenic impact. Significantly lower values of the length of the shoots, the diameter of the aerial part, and the length and number of leaves were recorded in the highlands of the SNP (CP 6) in the presence of anthropogenic impact (Figure 3a–e). Under undisturbed AR conditions, the parameters of the number of shoots per individual and potential seed productivity are significantly higher (Figure 3f–j).

### 2.2. Discussion of the Results of Morphometric Studies

We compared the morphometric parameters of *R. rosea* in our regions of research with the parameters of this species in other regions. It showed that in AR, individuals of this species were characterized by lower vigor compared to those studied in eastern Kazakhstan (EK) and in the south of the Ural Mountains (SU).

According to published data, in EK, the conditions on the floodplain meadows were optimal for the species [11]. This is where the species turned out to be the most productive. On the unshaded banks of mountain streams, specimens with mostly low (about 20 cm) shoots and large (5.5 cm in diameter) inflorescences were observed. The values of some morphometric parameters in individuals of this species in EK were characterized by a higher range of variability than those from individuals in AR. In EK, the length of the shoots was 24.4–49.3 cm, and the inflorescence was 3.2–5.5 cm in diameter. The development of more robust individuals than in AR was also observed. However, in EK, this species displayed lower competitive ability in the communities. The authors considered this feature to be the main limiting factor for the dispersal of *R. rosea*. Populations of this species migrate to areas free from turf vegetation or fall out of phytocenosis. In such habitats, the death of young plants of this species and low seed renewal is observed [11].

A decrease in the morphometric parameters of shoots was observed in SU as the absolute growth altitude of this species increased. The length of generative shoots ranged, on average, from 4.5 to 54.0 cm, and the number of generative shoots ranged from 1 to 38 pieces (on average, 5.5). These indicators turned out to be somewhat lower than in the individuals in AR (Table 2). In this region, according to the authors, *R. rosea* was subjected to severe stress associated with anthropogenic impact (unregulated harvesting of rhizomes) [10].

A wider range of variation in the morphometric characteristics of *R. rosea* than that found in our study was found in the Murmansk region (European part of Russia) [21]. This could be partially explained, in addition to natural causes, by the selection of only mature females in our study.

In the AR, *R. rosea* specimens in CP 1 and CP 2 turned out to be the most vigorous and productive, growing under favorable conditions in the subalpine meadows of the Katunsky Reserve. They also exhibited the highest potential seed productivity compared to plants in other ecological and geographical conditions of AR.

In the high-mountain conditions of the Kosh–Agach region (SNP, CP 6), individuals of this species were characterized by the smallest values of several morphometric features of the aerial part. In alpine locations, plants are affected by extreme weather conditions that contribute to the development of dwarfism even in species that are not elements of the alpine flora. In addition to the climatic factor, the productivity of *R. rosea* in this CP could be adversely affected by grazing cattle (yaks). According to the SNP records, in CP 6, grazing continues during the growing season, and the cattle summer camp is also located here. In the highlands of the Kosh–Agach region, this method of animal husbandry is common [22]. The negative impact of grazing is manifested in changes to the soil cover and the deterioration of the physical properties of the soil. This is demonstrated in a model that shows compaction, aeration reduction, and other soil cover indicators’ changes depending on the intensity, history, duration, and type of grazing [23]. We assume that the decrease in the size and productivity of *R. rosea* plants in CP 6 is associated not so much with high mountain conditions as with the impact of grazing.

This is confirmed by the fact that representatives from the highest mountain CP 3 (SNP) were mainly characterized by intermediate size of the organs of the aerial part of the shoot, and only the length of the shoots and volume of foliage had the smallest values. There is no cattle grazing in this habitat.

At relatively low altitudes in CP 4 and CP 5, the conditions for *R. rosea* plants are quite favorable. This is due to their growth in open areas with constant soil moisture from running water from melting snowfields. The morphometric parameters of the above-ground part of the shoots were mostly of intermediate values. However, in the presence of anthropogenic impact on CP 5, as well as in CP 6 in the SNP during grazing, *R. rosea* showed the lowest values of potential seed productivity.

In representatives from the ANR (CP 7) under natural conditions, we recorded the smallest length of shoots, as well as in the representatives from two other high-mountain CPs (3 and 6). The small length of shoots and the associated decrease in the photosynthetic surface was compensated by a significantly higher number of shoots due to an increase in the number of generative shoots in an individual. The latter reached the same high values as those of representatives from CP 1 and CP 2, located in protected areas in the KNR.

Similar transformations were observed by us in the subalpine species *Hedysarum austrosibiricum* Ledeb. and also in the species *Astragalus mongholicus* Bunge (Fabaceae), which has a wide altitudinal range in the highlands of the AR. In those species, the size of leaflets of a compound leaf decreased with an increase in the absolute altitude of growth. Such a decrease in photosynthetic surface in high-mountain CPs in representatives of *H. austrosibiricum* was compensated by the formation of a higher number of generative shoots and in vegetative shoots in individuals of *A. mongholicus* [24,25].

In *Rhaponticum carthamoides* (Willd.) Iljin. (Asteraceae), under conditions of grazing and harvesting of the underground parts of plants, we also observed a decrease in the thickness of the renewal zone and a reduction in the photosynthetic surface. However, these processes were accompanied by the preservation of a stable productivity of generative organs, which is comparable to their development in undisturbed habitats. Thus, *R. carthamoides* retained its reproductive potential under anthropogenic impact [26]. However, in *R. rosea* under conditions of grazing on high mountains in CP 6, only an uncompensated decrease in the parameters of the photosynthetic surface and potential seed productivity was observed, which may indicate the loss of resistance of individuals of this species under such extreme conditions for survival.

In addition, a significantly greater number of shoots in individuals from the ANR (in 2018) could have been caused by weather conditions. According to published weather data, 2018 was characterized by an early and prolonged spring, with a predominance of anomalous heat at the beginning of the season and an excess of moisture at the beginning and end of the season. The summer of 2018 was abnormally hot and dry [27]. The spring of 2020 was characterized by the predominance of anomalous heat and a lack of precipitation over most of the territory. At the beginning of the summer of the same year, cool weather with evenly distributed precipitation prevailed, but with showers and snow in the highlands [28]. It is possible that sufficient moisture at the beginning of the growing season in 2018 was favorable for the formation of more robust *R. rosea* individuals in the ANR in terms of the number of shoots (CP 7).

Therefore, the complex of growth conditions at a certain absolute altitude, the meteorological conditions of the year of observation, and anthropogenic impact had a significant effect on the variability of the main indicators of productivity in *R. rosea*. These basic parameters determine the size of the photosynthetic surface of plants of a given species and their reproductive potential. Under conditions of a dry beginning of the growing season and in the presence of anthropogenic impact (grazing and trampling), the productivity and sustainability of *R. rosea* significantly decreases.

### 2.3. Results of Genetic Analysis and Their Discussion

#### 2.3.1. Results of Genetic Analysis

**Figure 4:**
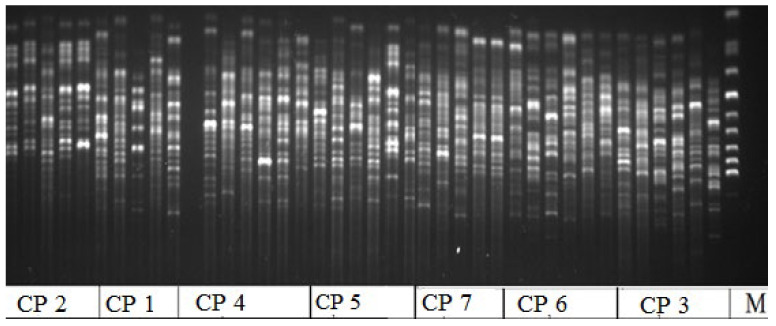
Electrophoregram of variability of the ISSR spectra distribution in *Rhodiola rosea* cenopopulations (CP) obtained with HB-12 primer; M is a marker; CP 1–CP 7 is a Nomber (No) of cenopopulation.

In terms of genetic polymorphism in *Rhodiola rosea* in the AR, individuals from the studied CPs were characterized by different levels of intrapopulation diversity. Based on the analysis of DNA fragments obtained via amplification with the selected ISSR primer, we observed the absence of genetic intrapopulation diversity in representatives of CP 3 and CP 6 (SNP). Representatives from these CPs also had the lowest number of effective alleles and a low Shannon index, which are the parameters that show the complexity of the population structure and allow for assessing the richness of the studied population (Table 3, the parameters). The electrophoregram obtained with primer HB-12 shows that representatives from CP 1 and CP 4 exhibited the greatest genetic diversity. They grew in the KNR (CP 1, CP 2) and at the base of Mount Krasnaya (CP 4) (Figure 4). They were characterized by the highest values of the number of effective alleles, the Shannon index, and the proportion and number of polymorphic loci (see Table 3).

**Figure 5:**
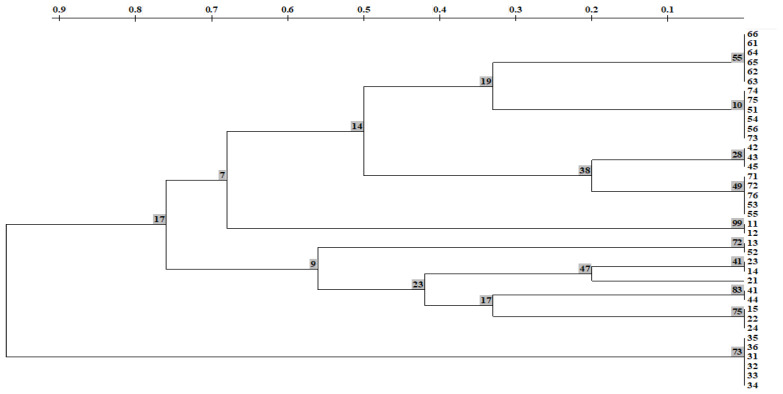
A dendrogram of genetic distances (HB-12) in *Rhodiola rosea* according to ISSR spectra based on the method of pairwise unweighted clustering using the TREECON program. In the nodes, the bootstrap support values are specified. The upper scale denotes genetic distances [30].

The UPGMA dendrogram, built using the Nei coefficient [29], shows the genetic distance between samples, varying approximately from 0.20 to 0.76. This is quite a high level of genetic similarity between samples from all studied population. On this dendrogram of the *R. rosea* populations, 3 clusters were formed in accordance with the studied individuals; branch nodes have high support (bootstrap index > 50%) (Figure 5).

Samples from CPs no. 1, 4, 5, 6, and 7 formed the first cluster. Samples from four CPs formed the second cluster: CP 1, CP 2, CP 4, and CP 5. Notably, individuals from CP 6 (SNP) on the dendrogram are assigned only to the first cluster, where they formed a separate clade. Even more genetically isolated than the representatives from CP 6 are representatives from CP 3 (SNP). Its individuals formed a separate cluster (Figure 5).

**Figure 6:**
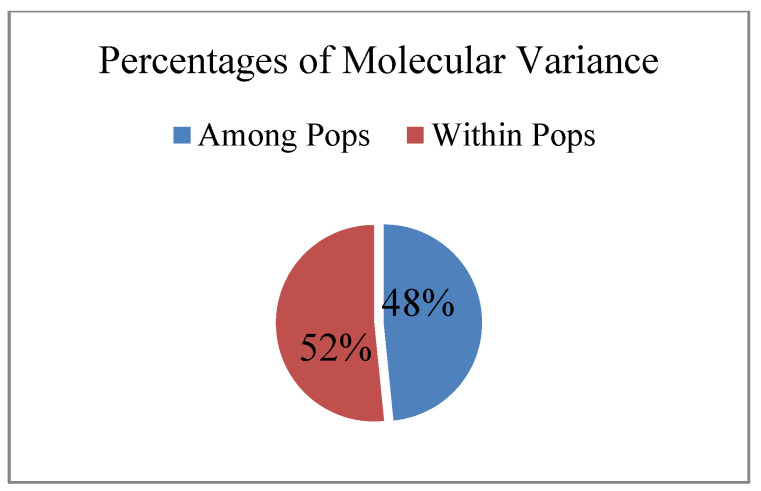
The ratio of intrapopulation and interpopulation variability in *Rhodiola rosea* in Altai Republic. Results of ANOVA-analysis of molecular variance (%).

According to ANOVA analysis, the proportion of intrapopulation variability in the studied populations of *R. rosea* was approximately equal to the proportion of intraspecific genetic variability (Figure 6).

Representatives from the KNR (CP 1 and CP 2) were characterized by the smallest genetic distance. They grew in close proximity to each other, and they had almost complete similarity in the distribution of ISSR markers (Table 4 and Table 5). Also, plants from CP 1 and CP 4, separated from each other by a distance of about 50 km, were also quite similar. The analysis showed that individuals from two pairs of populations (CP 3–CP 6 and CP 3–CP 2) had the highest differences in the distribution of ISSR markers.

Representatives from CP 3 and CP 6 are located relatively close geographically in the SNP, and the distance between them is a little more than 10 km, while representatives from CP 3 and CP 2 are more than 150 km apart from each other (see Table 5).

#### 2.3.2. Discussion of the Results of Genetic Analysis

According to the literature, no gender-specific or population-specific primers were found in *R. rosea.* In northern Europe, the average percentage of band polymorphism for ISSR markers was reported as follows: for Sweden—83.8%, Greenland—94.6%, and Faroe Islands—48.7% [15]. When conducting research in the AR, it was observed that the proportion of polymorphic loci for each of the populations is specific and its mean value for the species did not exceed 50%. We identified relatively high genetic heterogeneity in individuals from CP 1 and CP 4, compared to the studied samples from other habitats. They have the highest number of effective alleles (Table 2). This is consistent with the published data on this species in eastern Kazakhstan [20]. Individuals from CP 1 grew at a relatively low altitude in the protected conditions of the KNR. Growing in a protected area, probably, is a favorable factor for the relatively high genetic diversity of *R. rosea* in this habitat. The high genetic diversity of individuals from CP 4 may be due to favorable climatic conditions in this habitat and the absence of anthropogenic impact.

UPGMA dendrograms clearly demonstrate significant genetic differentiation of CP 3, which grows in SNP. It is the highest of all the studied populations in AR. It is possible that the highest absolute altitude of this CP influenced a certain genetic isolation of its individuals. The lowest number of effective alleles in the two high-mountain populations (CP 3 and CP 6) and the absence of polymorphic loci can be explained by their growth in high-mountain conditions (more than 2400 masl). These conditions can affect the genetic heterogeneity of populations, since they are far from the ecological optimum for this species and are located close to the limits of the altitudinal range of its growth. Also, in the highlands, only adapted individuals can survive. Therefore, the genetic heterogeneity of populations here can be reduced. The existence of geographically relatively close to each other populations of a species with high genetic isolation in the SNP (CP 3 and CP 6) indicates the presence of certain barriers to the exchange of genetic information among representatives from high-mountain populations.

According to published data, *R. rosea* is characterized by obligate xenogamy. In central Russia, the pollinators of *R. rosea* under cultivated conditions are hover flies (*Syrphidae* Latreille) [31]. According to these data, a large group of species from this family (subfamily *Syrphinae*), closely associated with tundra and alpine biocenoses, lives in the high mountains of Altai. This group of insects does not occur in lower biocenoses and is considered a “typical high mountain species” [31]. Due to the presence of pollinating species, the exchange of genetic information between populations of *R. rosea* in high mountains is quite possible. Harsh climatic conditions in the highlands are not an obstacle to the survival of pollinating insects adapted to them. The lack of opportunity for gene exchange between such populations (in our case, representatives from CP 3 and CP 6) can be due to the small number of their individuals in each population, since in each of them there were no more than 10–15 mature generative plants subjected to pollination. In this regard, they are isolated in space. In addition, in high-mountain habitats, the species occurs in single populations, which may also be associated with anthropogenic impact (the removal and use of *R. rosea* rhizomes by the local people), since regulated economic activity is allowed for the local population in the SNP.

## 3. Materials and Methods

### 3.1. Material and Methods of Morphometric Studies

**Figure 7:**
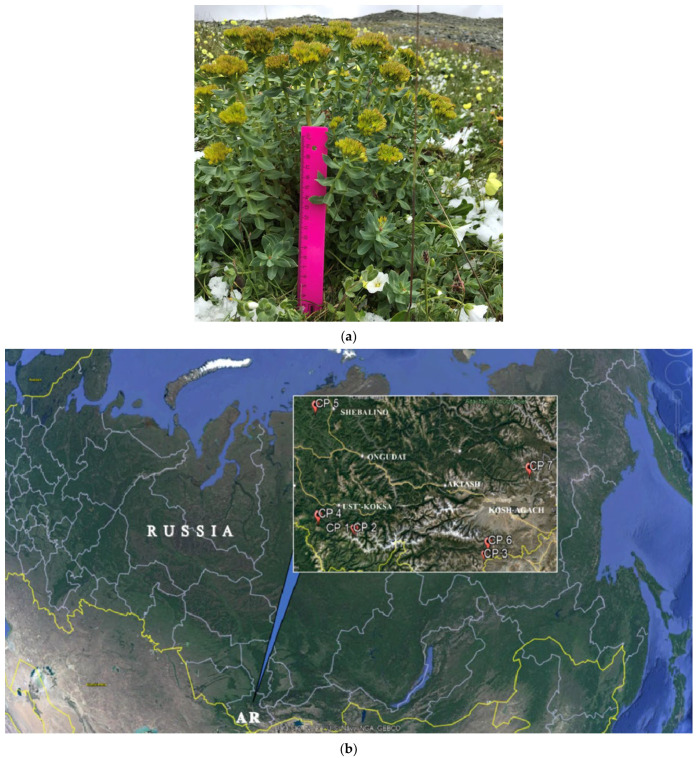
Female plant of *Rhodiola rosea* (**a**) and locations of the investigations of *Rhodiola rosea* cenopopulations in the Altai Republic (AR) (**b**). CP 1-CP 7 is a No of cenopopulations (see Table 6).

Live mature female *R. rosea* plants in the fruiting phase were studied in AR [32]. The climate of AR highlands is sharply continental. Minimum temperatures in winter reach −60 °C, and the period with stable negative temperatures continues for 8–9 months a year. The average annual temperatures in the regions vary from −4.2 °C to −8.5 °C. The amount of precipitation varies, on average ranging from 100 to 1000 mm per year [33]. Six cenopopulations (CP) were studied in 2020 (CP1–CP 6) and one in 2018 (CP 7). The range of study coordinates were 49–51° E and 85–89° N (Table 6 and Figure 7).

In each CP, the sample for morphometric studies was *n* = 15–21 (total 125) individuals. Potential seed productivity was studied in 78 individuals. According to our assumptions, the variability of quantitative traits of plants could be significantly influenced by abiotic factors: growth at different absolute altitudes (factor A) and weather conditions in different years of research (2018 and 2020) (factor B); as well as a biotic factor—anthropogenic impact (C).

We established the difference in absolute altitudes of 100 ± 50 m as a boundary for factor A. The CPs of *R. rosea* were studied at six altitudes: 1850, 2000, 2070, 2260, 2400, and 2500 m above sea level (Table 1).

Anthropogenic impact (C) was allotted to three gradations. In the Katunsky State Natural Biosphere Reserve (the KNR, CP 1 and CP 2), in Sailyugemsky National Park (SNP, CP 3), the Ust-Koksinsky District (vicinity of the village of Kaitanak, CP 4) and in the Altai Nature Reserve (ANR, CP 7), individuals of *R. rosea* grew under natural conditions (gradation 1). In the Ongudaysky district, on the Seminsky Pass (CP 5), individuals grew near the hiking trails. Due to easy accessibility, the rhizomes of *R. rosea* were harvested there in unregulated fashion for several years (gradation 2). The SNP (CP 6) is the location of a summer camp for the large herbivores (yaks), where regular grazing throughout the growing season is allowed by the regulating nature management authorities for representatives of the local population. This is ARea of the strongest anthropogenic impact (gradation 3).

Ten metric and allometric features of the aerial part of the shoot were assessed: the length of the shoots; diameter of the above-ground part of the plant; length and width of the leaf in the middle part of the generative shoot; diameter of inflorescence with fruits; number of leaves; total number of generative and vegetative shoots and their ratio; and leaf index (the ratio of the maximum to the minimum size). The parameters for the study of potential seed productivity (PSP) included: the diameter of inflorescence with fruits; number of flowers in inflorescence; number of carpels in the ovary; and potential seed productivity per generative shoot (the average value of the product of the total number of flowers and the number of carpels in the ovary on the generative shoot).

The names of taxa are given according to the original source [34]. Figure 1 shows sampling points with geographic coordinates displayed using the Google Earth web service [35].

### 3.2. Statistics of Morphometric Indicators

Variability and analysis of pairwise correlations (according to Spearman) were performed using Microsoft Excel software package 6.0. The mean value of the feature (M), standard error of mean (m), coefficient of variation (Cv, %), and correlation coefficient (r) were calculated. To reduce differences in dimension for correlation analysis, feature values are normalized according to the following equation: (Mi—M)/σ, where “Mi” is the feature value and “σ” is the standard deviation.

The variation was considered very low and low at values of the coefficients of variation (Cv) < 21% on the scale of C.A. Mamaev [36]. Variations above that were considered high. The nonparametric Kruskal–Wallis H-test in the StatSoft STATISTICA 6.0 software package program (Statsoft Inc.,Tulsa, OK, USA), was used to compare differences in the mean values of morphometric traits in the CPs (*n* < 25). Only the features with statistically significant differences (*p* < 0.05) in mean values are included in the discussion.

### 3.3. Materials and Methods of Genetic Research

The genetic polymorphism of *R. rosea* was studied in 7 CPs. The DNA from leaves dried under field and laboratory conditions (4–6 individuals from each CP) (without taking into account the gender of plants) was isolated via standard methods [37]. Eight primers were used: 884B, 98B, 99A, HB-12, M4, UBC835, UBC857, and UBC881. Of these, HB-12 was shown to be the most informative.

### 3.4. Statistics of Genetic Indicators

The results of statistical analysis and their visualization were analyzed with the TREECON software package 1.3b and the specialized macro GenAlEx6 for MS-Excel.

## 4. Conclusions

Comparison of the developmental features in seven *R. rosea* cenopopulations under different ecological and geographical conditions of the AR showed a higher vigor in individuals in undisturbed habitats in the middle mountains in the subalpine meadows of the KNR and the high mountains of the ANR. Here, individuals have the highest values of morphometric indicators of the aerial part, potential seed productivity, and high genetic heterogeneity, which reaches from 50 to 80%. In the undisturbed conditions of high mountains, *R. rosea* plants have adapted by increasing the number of generative shoots on average, 2.9 times. The most oppressed individuals of the species *R. rosea*, characterized by genetic homogeneity (lack of genetic polymorphism), grew in high-altitude conditions in the SNP under the constant summer grazing. Perhaps the combination of these two factors—growth in the highlands and constant grazing—is an extreme condition for the growth and development of *R. rosea*. Grazing is associated with negative impact: soil compaction on summer camp sites and concomitant damage to the above-ground parts of plants of this species. The impact of these unfavorable conditions on *R. rosea* plants can serve as a limiting factor for plant adaptation. Conservation of genetic diversity is a key factor for the survival of *R. rosea*. In the AR, the widespread unauthorized extermination of this species through harvesting of the underground part of plants is common. Therefore, in future research it is necessary to monitor the state of genetic diversity of as many natural populations of *R. rosea* as possible. First, their verification (certification) is necessary, and second, it is expedient to reintroduce them into natural conditions in order to preserve their genotypes in the territory of the AR. High—mountain populations, having a homogeneous genetic structure, are more vulnerable and need preservation. The study of various aspects of the variability of natural populations of *R. rosea* will enable the development of a sound program for conservation, restoration, and rational use of this rare species.

## Figures and Tables

**Table 1:** Variability of morphometric features of *Rhodiola rosea* plants in the Altai Republic.

Characteristics	Indicators	CP 1 N = 15	CP 2 N = 15	CP 3 N = 20	CP 4 N = 16	CP 5 N = 20	CP 6 N = 20	CP 7 N = 19	Total N = 125
1	M * ± m Cv,% min-max	47.2 ^a^ ± 2.2 18.9 31.7–64.0	44.4 ^a^ ± 3.4 30.1 4.5–60.5	21.3 ^b^ ± 1.0 25.6 13.0–35.0	23.4 ^ab^ ± 1.3 21.4 14.0–34.0	34.8 ^ab^ ± 1.5 19.9 25.0–48.0	20.7 ^b^ ± 0.8 22.3 13.0–32.0	28.6 ^b^ ± 1.1 18.0 17.0–37.5	30.8 ± 1.1 39.4 4.5–64.0
2	M ± m Cv, % min-max	26.8 ^a^ ± 2.9 42.5 12.0–57.0	19.4 ^a^ ± 2.5 49.2 6.0–39.0	8.3 ^ab^ ± 0.5 32.5 4.0–14.0	8.4 ^ab^ ± 0.8 36.7 4.0–14.0	7.5 ^ab^ ± 0.7 41.6 4.0–14.0	4.2 ^b^ ± 0.4 47.3 1.5–9.0	12.5 ^ab^ ± 1.1 37.7 6.0–26.5	11.9 ± 0.8 77.3 1.5–57.0
3	M ± m Cv, % min-max	2.7 ^ab^ ± 0.1 17.2 2.0–3.5	3.0 ^ab^ ± 0.1 16.6 2.0–4.0	2.4 ^ab^ ± 0.1 20.5 1.5–3.2	2.9 ^ab^ ± 0.2 28.5 1.5–4.6	2.8 ^ab^ ± 0.2 27.3 2.0–4.5	1.7 ^b^ ± 0.1 31.0 1.0–3.0	2.3 ^ab^ ± 0.1 19.0 1.7–3.5	2.5 ± 0.1 28.4 1.0–4.6
4 **	M ± m Cv, % min-max	1.0 ± 0.1 24.3 0.7–1.5	1.1 ± 0.1 19.8 0.8–1.5	0.9 ± 0.0 28.3 0.5–1.5	1.1 ± 0.1 23.6 0.7–1.6	1.0 ± 0.1 32.4 0.5–1.5	0.9 ± 0.1 44.8 0.5–1.8	0.88 ± 0.04 18.3 0.7–1.2	1.00 ± 0.01 29.1 0.5–1.8
5	M ± m Cv, % min-max	55.6 ^ab^ ± 6.8 48.8 3.0–116.0	81.3 ^a^ ± 4.8 22.7 58.0–132.0	27.2 ^b^ ± 0.8 16.3 19.0–34.0	49.0 ^ab^ ± 2.4 19.5 34.0–65.0	44.8 ^ab^ ± 2.3 23.5 22.0–63.0	35.9 ^ab^ ± 0.9 14.4 27.0–47.0	42.8 ^ab^ ± 1.6 16.3 29.0–55.0	47.0 ± 1.7 41.3 19.0–132.0
6	M ± m Cv, % min-max	10.8 ^a^ ± 2.2 80.9 2.0–32.0	11.9 ^ab^ ± 3.8 123.0 2.0–57.0	6.8 ^ab^ ± 1.0 82.4 1.0–26.0	7.3 ^ab^ ± 1.2 67.5 1.0–18.0	3.9 ^b^ ± 0.7 82.9 0–12.0	2.9 ^b^ ± 0.4 79.2 1.0–10.0	11.8 ^ab^ ± 2.8 106.2 0–40.0	7.8 ± 0.8 114.5 0–57.0
7	M ± m Cv, % min-max	5.3 ^ab^ ± 1.9 141.6 1.0–31.0	4.7 ^ab^ ± 1.0 78.0 1.0–14.0	5.5 ^ab^ ± 0.7 72.4 1.0–16.0	2.5 ^ab^ ± 0.4 61.2 1.0–5.0	1.9 ^b^ ± 0.2 51.9 1.0–4.0	2.2 ^ab^ ± 0.3 79.2 1.0–7.0	15.2 ^a^ ± 2.2 66.1 4.0–40.0	5.4 ± 0.6 125.6 1.0–40.0
8 **	M ± m Cv, % min-max	0.8 ± 0.3 150.8 0.1–4.5	1.3 ± 0.5 159.9 0.1–7.0	1.5 ± 0.4 106.3 0–5.3	0.5 ± 0.1 112.0 0.1–2.0	1.0 ± 0.2 110.4 0.1–4.0	0.9 ± 0.2 87.8 0.2–3.5	3.6 ± 1.6 159.3 0.19–20.0	1.4 ± 0.2 173.1 0.04–20.0
9	M ± m Cv, % min-max	16.1 ^a^ ± 3.6 89.3 5.0–57.0	16.6 ^a^ ± 3.8 88.4 4.0–60.0	12.3 ^ab^ ± 1.2 43.4 5.0–27.0	9.8 ^ab^ ± 1.5 62.7 3.0–22.0	5.7 ^b^ ± 0.7 56.7 2.0–14.0	4.6 ^b^ ± 0.8 72.8 2.0–16.0	33.2 ^a^ ± 4.6 49.7 15.0–58.0	13.0 ± 1.1 95.4 2.0–60.0
10	M ± m Cv, % min-max	2.8 ^ab^ ± 0.2 27.6 1.8–4.3	2.9 ^ab^ ± 0.2 20.9 2.0–4.3	2.7 ^ab^ ± 0.2 27.7 1.7–5.0	2.6 ^ab^ ± 0.1 17.8 1.7–3.4	3.1 ^ab^ ± 0.2 35.4 1.9–6.6	2.1 ^b^ ± 0.2 31.8 0.8–3.0	2.7 ^ab^ ± 0.1 18.0 1.8–3.5	2.7 ± 0.1 28.9 0.8–6.6

Legend: * M—mean value; m—error of the mean; Cv, %—coefficient of variation; min/max—minimum/maximum values. **: intermediate average values do not differ significantly. Superscripts: “^a^” and “^b^”—significant differences in mean values; “^ab^”—intermediate average value that does not differ significantly from the values indicated by “^a^” or “^b^”. Designations and dimension of features (cm—centimeters): 1—length of shoots; 2—diameter of the aerial part of the plant; 3—length of leaves; 4—width of leaf; 5—number of leaves; 6—number of vegetative shoots; 7—number of generative shoots; 8—the ratio of the number of generative to vegetative shoots; 9—total number of shoots; 10—leaf index: ratio of max/min size.

**Table 2:** Variability of some potential seed productivity characteristics of *Rhodiola rosea* in the Altai Republic.

Characteristics	Indicators	CP 1 N = 10	CP 2 N = 15	CP 3 N = 15	CP 4 N = 17	CP 5 N = 4	CP 6 N = 4	CP 7 N = 13	Total N = 78
Id, ** sm	M * ± m Cv, % min-max	2.9 ± 0.2 32.2 1.0–5.0	2.5 ± 0.3 44.4 1.0–5.0	1.9 ± 0.1 26.3 1.1–3.0	2.5 ±0.1 19.2 1.5–3.0	2.4 ± 0.2 23.8 1.5–3.3	2.1 ± 0.2 37.3 1.0–4.0	2.4 ± 0.1 32.5 1.0–5.0	2.2 ± 0.1 40.7 0.7–5.0
Nf	M ± m Cv, % Min-max	46.7 ^ab^ ± 4.0 26.9 33.0–71.0	66.2 ^a^ ± 9.1 53.4 20.0–139.0	35.3 ^ab^ ± 3.5 38.3 18.0–58.0	43.1 ^b^ ± 3.5 33.4 22.0–79.0	23.5 ^ab^ ± 4.7 40.1 15.0–37.0	28.3 ^ab^ ± 4.0 28.1 20.0–39.0	55.7 ^a^ ± 4.3 27.7 31.0–81.0	46.8 ± 2.6 49.0 15.0–139.0
Nc **	M± m Cv, % Min-max	4.1 ± 0.0 3.4 4.0–4.4	4.3 ± 0.1 6.5 4.0–4.6	4.0 ± 0.0 1.7 4.0–4.2	4.3 ± 0.1 8.7 3.8–5.0	3.5 ± 0.3 16.5 3.0–4.0	3.5 ± 0.3 16.5 3.0–4.0	4.3 ± 0.1 9.0 4.0–5.0	4.10 ± 0.04 9.5 3.0–5.0
PSP	M ± m Cv, % Min-max	189.9 ^ab^ ± 15.4 25.6 132.0–284.0	283.0 ^a^ ± 40.5 55.4 88.0–639.4	142.8 ^ab^ ± 14.6 39.7 72.0–243.6	185.4 ^ab^ ± 15.2 33.8 88.0–316.0	85.0 ^b^ ± 22.5 52.8 45.0–148.0	101.5 ^b^ ± 21.1 41.6 60.0–156.0	235.3 ^a^ ± 16.2 24.8 155.0–324.0	195.4 ± 11.5 101.2 45.0–639.4

Legend: * M—mean value; m—error of the mean; Cv, %—coefficient of variation; min/max—minimum/maximum values. Superscripts: “^a^” and “^b^”—significant differences in mean values; “^ab^”—intermediate average value that does not differ significantly from the values indicated as “^a^” or “^b^”. Id—diameter of inflorescence with fruits; Nf—number of flowers in inflorescence with fruits; Nc—number of carpels in one flower; PSP—potential seed productivity. **: intermediate average values do not differ significantly.

**Table 3:** Genetic diversity parameters in populations of *Rhodiola rosea*.

CP No	Na	Ne	I	h	uh	P
	M ± m	M ± m	M ± m	M ± m	M ± m	M ± m
CP 1	1.70 ± 0.33	1.69 ± 0.16	0.53 ± 0.11	0.37 ± 0.08	0.47 ± 0.09	83.33
CP 2	1.17 ± 0.40	1.43 ± 0.20	0.32 ± 0.15	0.23 ± 0.10	0.31 ± 0.14	50.00
CP 3	0.17 ± 0.16	1 ± 0	0	0	0	0
CP 4	1.67 ± 0.33	1.77 ± 0.15	0.56 ± 0.11	0.40 ± 0.08	0.50 ± 0.10	83.33
CP 5	1.00 ± 0.44	1.36 ± 0.18	0.29 ± 0.14	0.20 ± 0.09	0.24 ± 0.11	50.00
CP 6	0.33 ± 0.21	1.00 ± 0.01	0	0	0	0
CP 7	0.83 ± 0.40	1.33 ± 0.21	0.23 ± 0.15	0.17 ± 0.10	0.2 ± 0.12	33.33
M	0.98 ± 0.15	1.37 ± 0.07	0.28 ± 0.05	0.20 ± 0.04	0.25 ± 0.05	42.90

Designations: M—mean value; m—standard error of mean; P—proportion of polymorphic loci (%); Na—number of different alleles; Ne—number of effective alleles; I—Shannon information index; h—diversity; uh—impartial variety.

**Table 4:** Genetic distances between populations of *Rhodiola rosea* in the Altai Republic (Nei Genetic Distance).

CP No	Pairwise Population Matrix of Nei Unbiased Genetic Distance						
	CP 1	CP 2	CP 3	CP 4	CP 5	CP 6	CP 7
CP 1	0.000	-	-	-	-	-	-
CP 2	0.000	0.000	-	-	-		
CP 3	0.418	0.799	0.000	-	-		
CP 4	0.000	0.181	0.187	0.000	-	-	-
CP 5	0.103	0.317	0.448	0.000	0.000	-	-
CP 6	0.224	0.431	0.693	0.381	0.265	0.000	-
CP 7	0.173	0.399	0.582	0.000	0.000	0.294	0.000

**Table 5:** Geographic distances (km) between populations of *Rhodiola rosea* in the Altai Republic.

	CP 1	CP 2	CP 3	CP 4	CP 5	CP 6	CP 7
CP 1	0	-	-	-	-	-	-
CP 2	2.6	0	-	-	-	-	-
CP 3	171.1	173.7	0	-	-	-	-
CP 4	50.8	48.2	221.7	0	-	-	-
CP 5	150.5	149.2	280.2	128.6	0	-	-
CP 6	175.3	177.9	12.5	225.8	278.4	0	-
CP 7	237.3	239.6	112.9	282.5	291.6	100.4	0

**Table 6:** Habitat characteristics of *Rhodiola rosea* in the Altai Republic.

CP No	Location, Year of Research	Location Altitude (m Above Sea Level); Slope Exposure, Coordinates	Phytocenosis; Surrounding Plant Species
1.	Ust’-Koksinskij district, Katunskij nature reserve (KNR), lake shore Poperechnoe, 2020	1998 m; southwestern slope, 49.921111° N; 85.889444° E	Subalpine meadow; *Aconitum septentrionale* Koelle, *Betula rotundifolia* Regel & Tiling, *Carex* sp., *Chamaenerion angustifolium* (L.) Scop., *Cotoneaster uniflorus* Bunge *Dianthus superbus* L., *Poa pratensis* L., *Veratrum lobelianum* Bernh., *Hedysarum neglectum* Ledeb., *Rumex confertus* Willd., *Solidago virgaurea* L., *Salix* sp.
2.	Ust’-Koksinskij district, the KNR, water flow between lakes Suroch’e and Verhnee Mul’tinskoe, 2020	2070 m; western slope, 49.928056° N; 85.854722° E	Subalpine meadow; *Poa pratensis* L., *Veratrum lobelianum* Bernh., *Hedysarum neglectum* Ledeb., *Rumex confertus* Willd., *Solidago virgaurea* L., *Aconitum septentrionale* Koelle, *Salix* sp., *Saussurea alpina* (L.) DC., *Sanguisorba alpina* Bunge, *Delphinium laxiflorum* DC.
3.	Kosh-agachskij district; Sailyugemsky National Park (SNP), along the watercourse river Usaj	2522 m; 49.456944° N; 88.156667° E	Mountain tundra; *Dryas oxyodonta* Juz., *Rumex* sp., *Ranunculus* sp., *Dasiphora parvifolia* (Fisch.) Juz., *Dasiphora parvifolia* (Fisch.) Juz., *Galium* sp., *Potentilla* sp., *Aconitum* sp., *Gentiana algida* Pall., *Betula rotundifolia* Spach, *Artemisia* sp., *Swertia obtusa* Ledeb., *Cotoneaster* sp., *Rhodiola rosea* L., *Spiraea alpine* Pall., *Rhodiola coccinea* (Royle) Boriss.
4.	Ust’-Koksinskij district, vicinity of the village Kajtanak, slope of Krasnaja Mountain, 2020	2000 m; rocky southeastern slope; glacial lake shore 50.076944° N; 85.221667° E	Sparse cedar forest; *Betula rotundifolia* Spach; *Dryas oxyodonta* Juz.; *Salix KNRylovii* E. Wolf.; *Salix reticulata* L.; *Aquilegia sibirica* Lam.; *Bergenia crassifolia*; *Bistorta vivipara* (L.) Delarb.; *Carex melanantha* C.A. Mey.; *Doronicum altaicum* Pall.; *Dracocephalum grandiflorum* L.; *Hedysarum austrosibiricum* B. Fedtsch.; *Oxyria digyna* (L.) Hill.; *Platanthera bifolia* (L.) Rich.; *Potentilla gelida* C.A. Mey.; *Ranunculus altaicus* Laxm.; *Rhodiola rosea* L.; *Swertia obtusa K. Koch*; *Trollius asiaticus* L.; *Viola altaica* L.
5.	Ongudajskij district, Seminskij pass, 2020	1850 m; northern slope; 51.230833° N; 85.351944° E	Sparse cedar forest; *Rhaponticum carthamoides* (Willd.) Iljin, *Geranium albiflorum* Ledeb., *Bupleurum aureum* Fisch. ex Hoffm., *Poa palustris* L., *Trollius altaicus* C.A. Mey., *Hedysarum neglectum* Ledeb.
6.	Kosh-agachskij district; SNP shore of lake Tunguryuk, 2020	2412 _M_; eastern slope; 49.541389° N; 88.256111° E	Grain-grass forb meadow; *Ligularia altaica* DC., *Achillea asiatica* Serg., *Spiraea alpina* Pall., *Dasiphora parvifolia* (Fisch.) Juz., *Gentiana macrophylla* Pall., *Antennaria dioica* (L.) Gaertn., *Achillea* sp., *Veronica* sp., *Potentilla argentea* L., *Dracocephalum grandiflorum* L., *Rhodiola rosea* L., *Leontopodium ochroleucum* Beauverd
7.	Ulagansky district, territory of the Altai Nature Reserve (ANR) in the valley of the Bogoyash River, 2018	2260 m; 50.2281° N; 89.1807° E	Mountain tundra; *Achillea asiatica*, *Bistorta vivipara*, *Gentiana frigida* Haenke, *Gentiana pneumonante* L., *Hedysarum consanquineum* DC., *Phlojodicarpus sibiricus* (Fisch. ex Spreng.) Koso-Pol., *Poa palustris, Potentilla parvifolia,* *Rumex acetosa*, *Rhodiola algida, Saxifraga hirculus* L., *Schulzia crinita* (Pall.) Spreng., *Spiraea alpina* Pall., *Trollius altaicus*, *Campanula rotundifolia* L.

## Data Availability

The data presented in this study are available upon request from the corresponding author. The data are not publicly available due to privacy.
